# Introducing Region Based Pooling for handling a varied number of EEG channels for deep learning models

**DOI:** 10.3389/fninf.2023.1272791

**Published:** 2024-01-30

**Authors:** Thomas Tveitstøl, Mats Tveter, Ana S. Pérez T., Christoffer Hatlestad-Hall, Anis Yazidi, Hugo L. Hammer, Ira R. J. Hebold Haraldsen

**Affiliations:** ^1^Department of Neurology, Oslo University Hospital, Oslo, Norway; ^2^Institute of Clinical Medicine, Faculty of Medicine, University of Oslo, Oslo, Norway; ^3^Department of Computer Science, Oslo Metropolitan University, Oslo, Norway; ^4^Department of Holistic Systems, SimulaMet, Oslo, Norway

**Keywords:** EEG, deep learning, machine learning, cross-dataset, cross-channel system, convolutional neural networks, time series, Region Based Pooling

## Abstract

**Introduction:**

A challenge when applying an artificial intelligence (AI) deep learning (DL) approach to novel electroencephalography (EEG) data, is the DL architecture's lack of adaptability to changing numbers of EEG channels. That is, the number of channels cannot vary neither in the training data, nor upon deployment. Such highly specific hardware constraints put major limitations on the clinical usability and scalability of the DL models.

**Methods:**

In this work, we propose a technique for handling such varied numbers of EEG channels by splitting the EEG montages into distinct regions and merge the channels within the same region to a region representation. The solution is termed *Region Based Pooling* (RBP). The procedure of splitting the montage into regions is performed repeatedly with different region configurations, to minimize potential loss of information. As RBP maps a varied number of EEG channels to a fixed number of region representations, both current and future DL architectures may apply RBP with ease. To demonstrate and evaluate the adequacy of RBP to handle a varied number of EEG channels, sex classification based solely on EEG was used as a test example. The DL models were trained on 129 channels, and tested on 32, 65, and 129-channels versions of the data using the same channel positions scheme. The baselines for comparison were zero-filling the missing channels and applying spherical spline interpolation. The performances were estimated using 5-fold cross validation.

**Results:**

For the 32-channel system version, the mean AUC values across the folds were: RBP (93.34%), spherical spline interpolation (93.36%), and zero-filling (76.82%). Similarly, on the 65-channel system version, the performances were: RBP (93.66%), spherical spline interpolation (93.50%), and zero-filling (85.58%). Finally, the 129-channel system version produced the following results: RBP (94.68%), spherical spline interpolation (93.86%), and zero-filling (91.92%).

**Conclusion:**

In conclusion, RBP obtained similar results to spherical spline interpolation, and superior results to zero-filling. We encourage further research and development of DL models in the cross-dataset setting, including the use of methods such as RBP and spherical spline interpolation to handle a varied number of EEG channels.

## 1 Introduction

Recent advancements in artificial intelligence (AI) have opened up new opportunities for the fields of cognitive neuroscience and clinical brain health research. In this context, the EU Horizon 2020 funded project AI-Mind (www.ai-mind.eu) has been established, which aims at developing AI-based tools to estimate the risk of dementia for people affected by mild cognitive impairment. The project collects a comprehensive set of biomarkers, including blood samples, sociodemographic information, digital cognitive test scores, and electroencephalography (EEG) data. A combination of traditional machine learning (ML) and deep learning (DL)-based algorithms will be employed. While the former commonly provides improved transparency and integration of domain knowledge, the latter has the capacity to find patterns and extract features in complex and unstructured data beyond what can be obtained by hand-crafted features.

DL is a method in AI with potential to significantly transform healthcare services (Hinton, 2018). By processing data in multiple layers, DL learns representations with different levels of abstraction. Breakthroughs of DL include processing of images, video, speech, audio, and text (LeCun et al., 2015). Despite the progress in research and development, there are still significant gaps to be filled for deployment of AI in clinical practice, such as mitigating discriminatory bias and improving generalization to new populations (Kelly et al., 2019; Chen et al., 2023). In particular, AI systems trained on datasets with an underrepresentation of marginalized groups have an elevated risk of bias toward those groups (Rajpurkar et al., 2022). Furthermore, AI algorithms trained on data generated by a single system (e.g., when all imaging data are collected using the same camera with fixed settings) may exhibit single-source bias, resulting in a decrease in performance on inputs collected from other systems (Rajpurkar et al., 2022). For the AI-Mind project, such biases may pose challenges requiring particular considerations. While about two-thirds of dementia cases are in low-income and middle-income countries (LMICs), extrapolating predictive models developed in high-income countries to LMICs is not always feasible (Stephan et al., 2020). A technical prerequisite for extrapolating models to LMICs is the availability of hardware needed for data acquisition. As a neuroimaging modality, EEG is low-cost and mobile compared to magnetic resonance imaging and magnetoencephalography. Moreover, it does not require a dedicated isolated room. Extrapolation of EEG biomarkers to LMICs is thus not hindered by difficulties in installation of the acquisition hardware.

The recent progress of DL has significantly increased its relevance for EEG data analysis (Roy et al., 2019). Domains of application include emotion recognition (Houssein et al., 2022), driver drowsiness (Stancin et al., 2021; Mohammed et al., 2022), classification of alcoholic EEG (Farsi et al., 2021), epileptic seizure detection (Ahmad et al., 2022), mental disorders (de Bardeci et al., 2021), schizophrenia (Oh et al., 2019), major depressive disorder and bipolar disorder detection (Yasin et al., 2021), motor imagery and other brain computer interface (BCI)-related problems (Lotte et al., 2018; Abo Alzahab et al., 2021). Despite the attention of DL in EEG, little research has focused on issues relating to the cross-dataset setting and generalization (Wei et al., 2022). As AI-Mind will use EEG signals for its algorithm development, enabling our tools for deployment on multiple data acquisition systems and mitigating discriminatory bias, is a necessity.

However, a common limitation of many existing DL architectures occurring specifically to EEG is their inherent inability to handle a varied number of channels as input data (Wei et al., 2022). This lack of compatibility conflicts with the real-world high variety of EEG hardware and hinders training and deployment on heterogeneous datasets where both the number of electrodes and their positions on the scalp may vary. Hence, this challenge not only prevents integration of DL models into diverse EEG setups but also limits the inclusion of larger sample sizes as well as more heterogeneous and representative data. Moreover, evidence from clinical neurology research suggests that the number of channels used during EEG recording may have a significant impact on the data's ability to capture spatially limited phenomena (Hatlestad-Hall et al., 2023). The inability to handle this diversity originates from tensors such as matrices and vectors requiring fixed dimensions to be compatible from a linear algebraic perspective. To address this technical issue, this work aims at introducing a simple methodological framework which can be used in combination with current and future DL models to handle a varied number of electrodes. Here, two methods for scaling the data to fit into the DL model are used as baselines for comparison: (1) zero-filling missing channels and (2) applying spherical spline interpolation (Perrin et al., 1989).

There exist several techniques which may leverage external datasets to improve DL models, which we hypothesize will play a significant role in cross-dataset learning and generalization. Approaches such as unsupervised and self-supervised learning may be utilized even in the absence of the target of interest. Improvements may be in terms of, e.g., performance or generalization, and are considered to play an important role for data efficiency of DL (Hinton, 2018; Hendrycks et al., 2019; Banville et al., 2021). In the field of EEG research, Kostas et al. (2021) obtained improved results on multiple downstream datasets by using contrastive self-supervised learning on a large dataset for pre-training. Furthermore, Banville et al. (2021) successfully applied self-supervised learning to sleep staging and pathology detection. Another approach on heterogeneous EEG datasets is to use transfer learning, shown in the BEETL competition (Wei et al., 2022). Furthermore, a desired outcome of AI-Mind is to characterize brain networks from EEG data. While metrics from neuroscientific literature have known cognitive relevance (Stam et al., 2006), a DL methodology to obtain features of similar neurophysiological meaning seems non-trivial. This is due to features of DL being *learned* in a data-driven manner rather than human defined to capture the underlying neurophysiological phenomena. We hypothesize, however, that feature learning and pre-trained models may be viable alternatives.

The intended purposes for developing methods for handling a varied number of channels with possibly different positions on the scalp are (1) to enable the application of DL models on a range of existing and varied EEG systems. For clinical implementation, a highly desired property is to have a method which works on the EEG systems currently in use at different clinical centers around the world. The number of channels and channel locations are indeed varied, meaning that it is a necessity to handle this diversity, to maximize outreach and clinical usefulness; (2) to be able to pre-train or perform representation learning on heterogeneous and large amounts of data. There are many open-source datasets from a range of nationalities, pathologies, age groups, and cohorts. To generalize across such data, methods including pre-training and representation learning on multiple and heterogeneous datasets may be a step in the right direction, as it can lead to more robust and generalized features. Improving the robustness and generalization may in turn improve the fairness and equity of the developed AI models. This relevance extends to all medical use and integration of DL in EEG, including the generation of synthetic data (Goodfellow et al., 2014) and digital twins (Grieves and Vickers, 2017), and enabling of simulation techniques for improved clinical treatment selection. Indeed, developing methods to facilitate the evolution of such precision medicine approaches is essential. This study does not carry out such pre-training or representation learning but introduces a framework for enabling it to be performed in a larger scale, with a varied number of electrodes. Instead, this study conducts an initial evaluation to ascertain the efficacy or inadequacy of the framework.

Our framework is designed to be model agnostic, meaning that both current and future DL architectures can apply it with ease. The code is publicly available and may be used to develop customized implementations of the framework, or to combine it with other DL architectures. Furthermore, we aim to experimentally demonstrate that by applying our framework, the algorithm performance in itself remains the same.

## 2 Materials and methods

**Figure 1:**
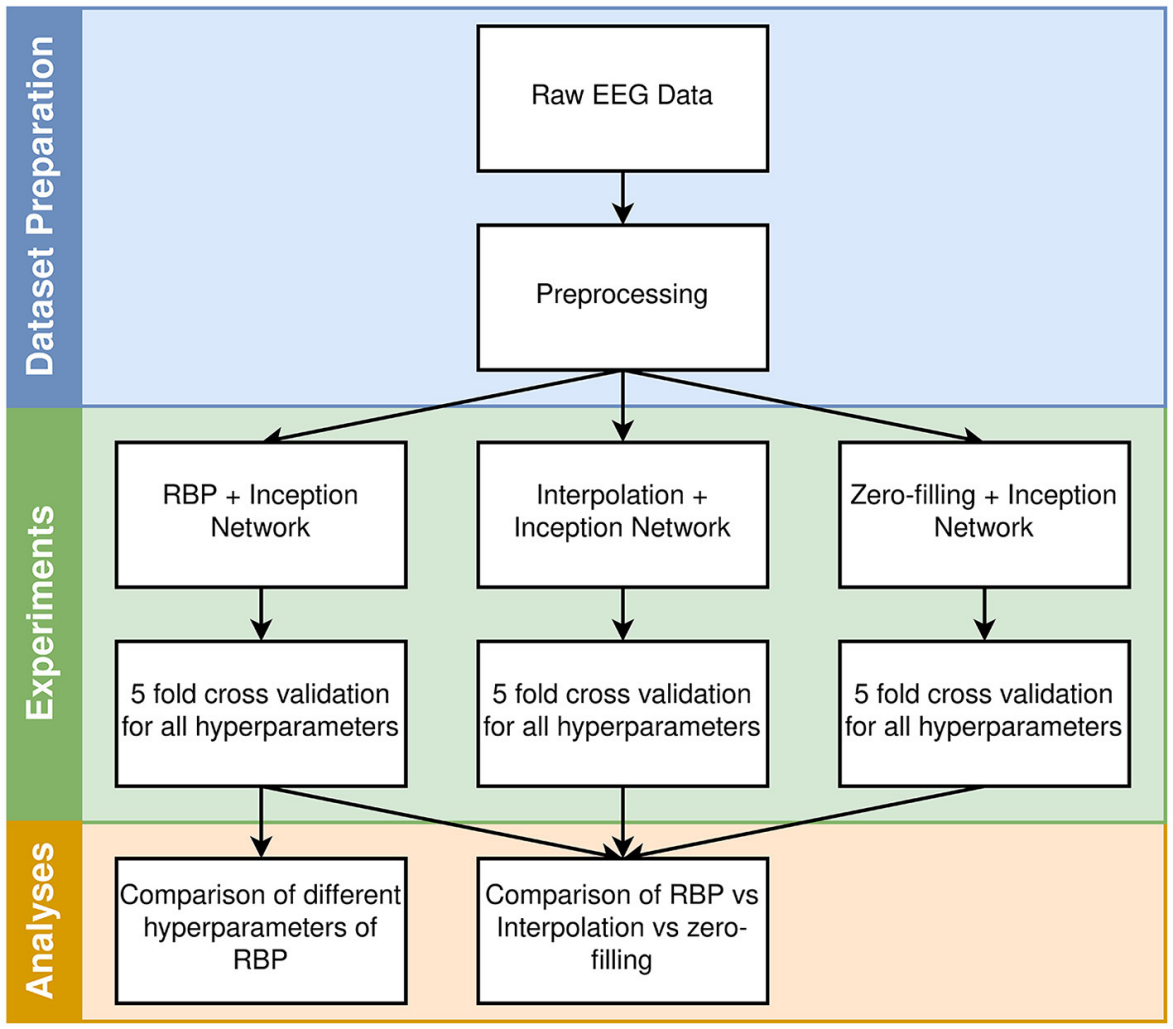
High-level overview of the workflow. The different hyperparameters for each model are described in Section 2.5.

In this section, the dataset, methods, models, and experiments are described. A high-level overview of the workflow is provided in Figure 1.

### 2.1 Data

The data used for this study is an open-source dataset from Child Mind Institute (Alexander et al., 2017). It contains a large high-density EEG (129 electrodes) dataset from the age distribution 5–21 years, including male and female subjects, with varied brain pathologies. The objective of the DL models was to classify the sex of a subject, given the EEG data. After removing samples which did not fulfill the inclusion criteria for data quality (see Section 2.1.1), the dataset was balanced by down sampling the class in abundance, resulting in a final dataset with 1,788 subjects. Only the resting-state EEG data files were extracted. The first 30 s of the recordings were skipped as the first parts of the EEG are more likely to contain unwanted artifacts. The proceeding 10 s was used as input for the models. Only a single 10 s window was used per subject, and the splitting of data was thus made on subject level. The sampling frequency was kept at 500 Hz as in the original dataset.

#### 2.1.1 Preprocessing

The raw data was preprocessed using an automated data cleaning pipeline developed in MATLAB, using functions from the EEGLAB toolbox (Delorme and Makeig, 2004). Channels with low-quality data were removed by iterative exclusion of signals with amplitude standard deviation *SD*>75μV or no amplitude variation at all. The EEG file was rejected if the number of excluded channels exceeded 39 (>30%). Line artifacts were removed with Zapline (de Cheveigné, 2020), and the signals were band-pass filtered between 1 and 45 Hz. Excluded channels were replaced with interpolated signals to ensure data dimension consistency. The channels were re-referenced to the average of all scalp channels. The pipeline is available at GitHub.^1^

### 2.2 Inception network

The Inception network is a convolutional neural network (CNN) based architecture, which is the main building block of InceptionTime. Here, the Inception network is briefly described, and for further details on the architecture, the reader is referred to the original study (Ismail Fawaz et al., 2020).

An Inception network is composed of multiple Inception modules, with linear shortcut connections for every third Inception module. A key component of the Inception module is the bottleneck layer, which effectively computes linear combinations of the input time series. Furthermore, the Inception module applies filters of different lengths simultaneously on the same input time series, and resulting feature maps are aggregated by concatenation. After passing the data through all Inception modules, global average pooling is performed in the temporal dimension. Finally, while the original Inception network used a fully connected layer with softmax activation, this was changed to a single fully connected layer with sigmoid activation (Ismail Fawaz et al., 2020).

The hyperparameters of our Inception network was set as described in the original study. This includes a depth of six Inception modules, and 32 number of filters for all convolutional kernels in all Inception modules (Ismail Fawaz et al., 2020).

### 2.3 Methods for handling a varied number of channels

Three methods for handling a varied number of channels were tested on a binary classification problem, sex prediction. The three methods were (1) zero-filling, (2) spherical spline interpolation (Perrin et al., 1989), and our suggested new method (3) Region Based Pooling (RBP). Inception network (Ismail Fawaz et al., 2020) was the DL model used after zero-filling, interpolation, or applying RBP, with the exception that the final layer used scalar output and sigmoid as activation function for predictions.

### 2.4 Region based pooling

**Figure 2:**
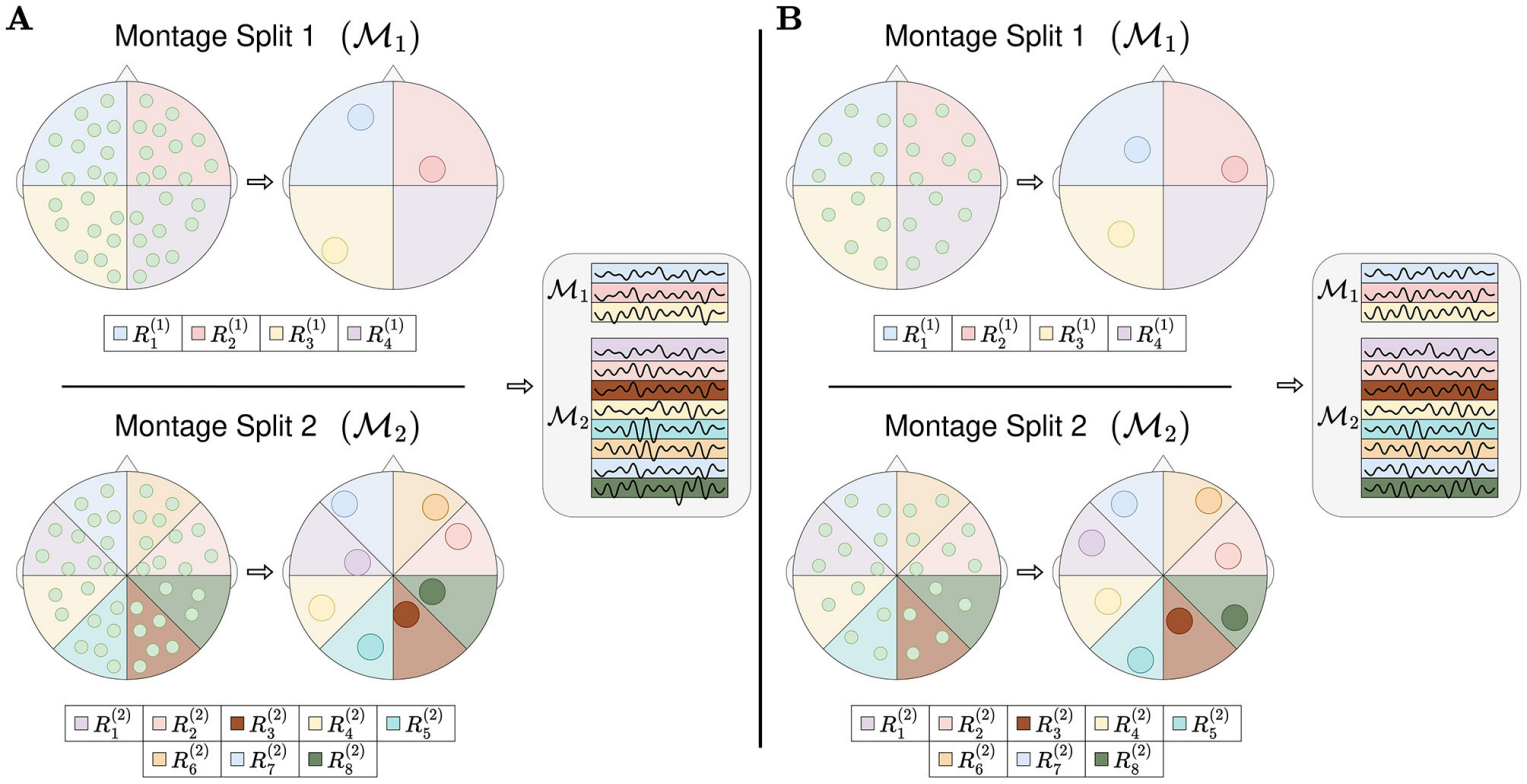
Region based pooling. The EEG montage is split into multiple regions. All channels in the same region are pooled into a region representation. Multiple montage splits may be performed, and the number of montage splits equals to two in this figure, $M_{1}$ and $M_{2}$. If there is at least one channel in all used regions, the mapping from channels into region representations can be made. This is illustrated as channel system A and channel system B have unequal numbers of channels with different channel locations, and they can both obtain region representations. After pooling channels into region representations, the region representations are stacked/row concatenated. The sequence of stacking represents an arbitrarily chosen design.

RBP splits the topology of the EEG montage into regions, as illustrated in Figure 2. The channels within a single region are pooled into one or more *region representations*, and hence the name *Region Based Pooling*. To minimize the loss of information, multiple splits with different region formations are performed. RBP introduces three new optimization problems; (1) how to split the EEG montage into regions (both the number of montage splits and the algorithm separating the regions), (2) how to pool the channels within the same region, and (3) how to merge the outputs of the different montage splits. The proceeding two subsections intend to illustrate how the first two problems can be addressed and are meant as examples of implementation.

All RBP models in the experiments of this study merged the outputs of the montage splits by concatenation. Furthermore, all channels within the same region were merged to a single region representation. Finally, all region representations were normalized by subtracting the mean and dividing by the standard deviation in the temporal dimension.

#### 2.4.1 Method for splitting into regions

A montage split is a region-based partitioning of the EEG montage. The set of all montage splits are denoted {$M_{1}$, $M_{2}$, ..., $M_{n}$}, where *n* is the number of montage splits. Each montage split contains multiple regions, $M_{i}=\left\{R_{1}^{\left(i\right)},R_{2}^{\left(i\right)},...,R_{m_{i}}^{\left(i\right)}\right\}\;\forall i\in \left\{1,...,n\right\}$, where the regions may or may not overlap. Furthermore, a montage split may or may not cover the entire EEG montage. Given a channel system *C* which is compatible with the partitioning, the *j*-th region of the *i*-th montage split $R_{j}^{\left(i\right)}\in M_{i}$ contains the channels $R_{j}^{\left(i\right)}⊃R_{j}^{\left(i\right)}\cap C$, where $R_{j}^{\left(i\right)}\cap C$ denotes the set of channels of channel system *C*, positioned within the boundaries of $R_{j}^{\left(i\right)}$.

**Figure 3:**
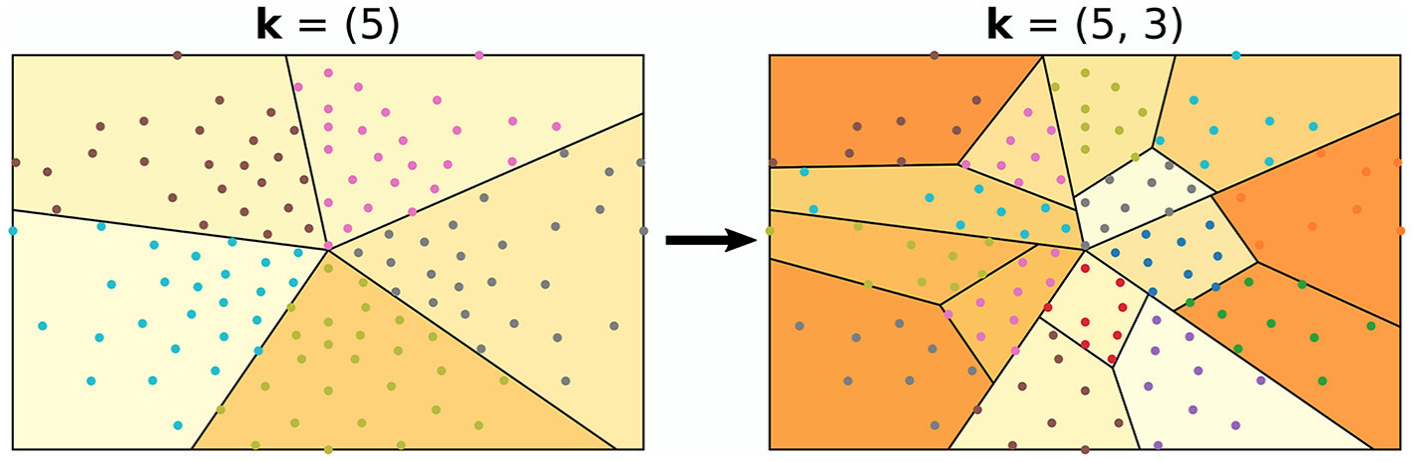
Example of how the EEG montage may be split into regions. In this example, the split vector was set to **k** = (5, 3). This can be observed, as the montage was first split into five regions, followed by splitting those into three regions.

The algorithm used in all experiments for splitting the montage into regions is illustrated in Figure 3. It follows an iterative procedure and was designed to not have overlapping regions. Furthermore, all regions are used for all montage splits. The algorithm requires one to fix a *split vector* $\text{k}={\left(k_{1},k_{2},...,k_{p}\right)}^{T}\in {\mathbb{N}}_{2}^{p}$, where the elements of **k** and *p* are design choices/hyperparameters. As a pre-step of the algorithm, all channel positions are mapped to 2D coordinates. Thereafter, the centroid of the channel positions is calculated, and a random angle is generated. With the centroid and the random angle as starting point and angle, *k*_1_−1 angles are computed such that the angles split the channels into *k*_1_ equally sized regions. Here, the size of a region refers to the number of channels within it. For all newly generated regions, the same procedure is repeated; (1) compute the centroid (2) generate a random angle, and (3) generate *k*_2_−1 angles such that *k*_2_ number of equally sized regions are formed. This iterative approach is executed either *p* times, or until the number of channels in the regions are too low, defined by a stopping criteria *min*_*nodes*.

For the experiments, there were seven different split vectors, **k** = (3, 3, 3)^*T*^, **k** = (4, 2, 4)^*T*^, **k** = (2, 4, 2)^*T*^, **k** = (2, 2, 2, 2, 2)^*T*^, **k** = (3, 2, 3)^*T*^, **k** = (2, 3, 2)^*T*^, and **k** = (3, 4, 2)^*T*^. For each montage split, the selection of **k** was made by random sampling with equal probabilities. The stopping criteria was one of the hyperparameters for grid search and included *min*_*nodes*∈{1, 2, 3}.

#### 2.4.2 Pooling operations

To enable compatibility with a varied number of channels with possibly different channel positions, defining pooling mechanisms which can input and handle multivariate time series of different dimensions within the regions, is a prerequisite. That is, to apply mechanisms within the regions which can map a varied number of channels to a single region representation. Finding sophisticated mechanisms with this property may be crucial for RBP. This subsection presents several approaches for pooling mechanisms.

##### 2.4.2.1 Average

The first pooling mechanism is to merge the channels within a region by computing its mean in channel dimension. This offers a simple and time-efficient method and aggregates the channels with equal contributions for computing region representations.

##### 2.4.2.2 Channel attention

**Figure 4:**
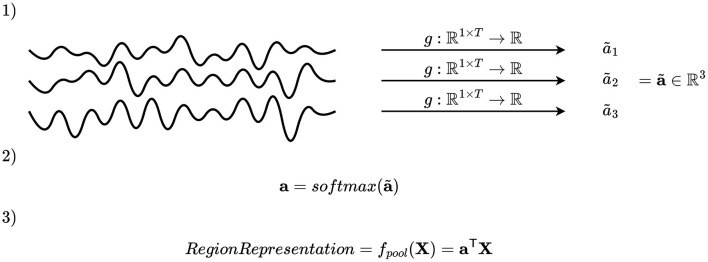
Illustration of channel attention mechanism. An importance scalar is computed for each channel, and the attention vector is computed by applying softmax on a concatenation of these. The elements of the attention vector are used as coefficients to compute a linear combination of the channels.

A second pooling mechanism is to select the key channels by first assigning an importance score, and secondly merge the channels by computing a weighted average based on the importance scores. Mathematically, this may be accomplished by defining a function *g*:ℝ^1 × *T*^ → ℝ, where *T* denotes the number of time samples, applied on all time series within the region, and using the values obtained to compute coefficients of a linear combination, as illustrated in Figure 4. Applying *g* to each channel in a region gives an importance scalar for each channel, which is subsequently concatenated and passed to a softmax activation function, giving the channel attention vector of the *i*-th montage split and *j*-th region ${\text{a}}^{\left(i,j\right)}\in \left\{\text{q}\in {\left(0,1\right)}^{\left|R_{j}^{\left(i\right)}\cap C\right|}:||\text{q}|{|}_{1}=1\right\}$. The vectors **a**^(*i, j*)^ have the properties that the entries are positive and sum to one due to the softmax activation function. After computing **a**^(*i, j*)^, the channels of the *i*-th montage split and *j*-th region are pooled by weighted averaging $f_{pool}^{\left(i,j\right)}\left(\text{X},C\right)={\text{a}}^{\left(i,j\right)T}{\text{X}}_{R_{j}^{\left(i\right)}\cap C}$, where ${\text{X}}_{R_{j}^{\left(i\right)}\cap C}\in {\mathbb{R}}^{|R_{j}^{\left(i\right)}\cap C|\times T}$ are the EEG time series of all channels within the region.

*ROCKET-based features*: Random Convolutional Kernel Transform (ROCKET) (Dempster et al., 2020) is a highly efficient time series classifier, which obtained high performance in a short time frame in a multivariate time series classification bake off (Ruiz et al., 2021). For feature extraction, ROCKET applies a large number of diverse, random and non-trainable convolutional kernels, and computes the proportion of positive values and maximum value of the resulting feature maps. This was adopted as a pooling mechanism, where the proportion of positive values and max values of the feature maps were used for computing the importance score of a channel. From the *num*_*kernels* · 2 features, a trainable fully connected module with scalar output and specific to the *i*-th montage split and *j*-th region, *FC*^(*i, j*)^:ℝ^*num*_*kernels*·2^ → ℝ, was applied. After computing the importance scores for all time series in the region, a softmax activation function was applied to obtain positive coefficients only, which sum to one. A desirable property of using non-trainable convolutional kernels is that the output feature maps (along with proportion of positive values and max values) are being computed only once per subject, prior to training. Therefore, the computational cost of a large number of convolutions may be justified by its property to be pre-computed.

The number of convolutional kernels was set to 1000, and the maximum receptive field in the temporal dimension to 250, which corresponds to half a second with the given sampling rate. This was based on computational feasibility, taking both time consumption and memory usage on limited hardware into account. Furthermore, no padding was used, in contrast to the original implementation. The ROCKET features were pre-computed prior to training, as the convolutional kernel weights were frozen, and the proportion of positive values and max values of the feature maps were thus constant per channel and subject during training. Furthermore, the ROCKET kernels were shared across all regions and montage splits to reduce runtime. The FC modules mapping the *num*_*kernels*·2 features to a single coefficient, used only a single fully connected layer with linear activation function. That is, for every subject, the importance score of the *k*-th channel in the *j*-th region of the *i*-th montage split prior to softmax normalization, was computed as $g^{\left(i,j\right)}\left({\text{x}}_{k}\right)=FC^{\left(i,j\right)}\left({\text{z}}_{k}\right)={\text{w}}_{i,j}^{T}{\text{z}}_{k}$, where ${\text{w}}_{i,j}\in {\mathbb{R}}^{num\text{\_}kernels\cdot 2}$ is a trainable weight vector of the *j*-th region of the *i*-th montage split, ${\text{x}}_{k}\in {\mathbb{R}}^{T}$ is the time series of the *k*-th channel, and ${\text{z}}_{k}\in {\mathbb{R}}^{num\text{\_}kernels\cdot 2}$ is the pre-computed ROCKET features of channel *k*.

##### 2.4.2.3 Continuous channel attention

**Figure 5:**
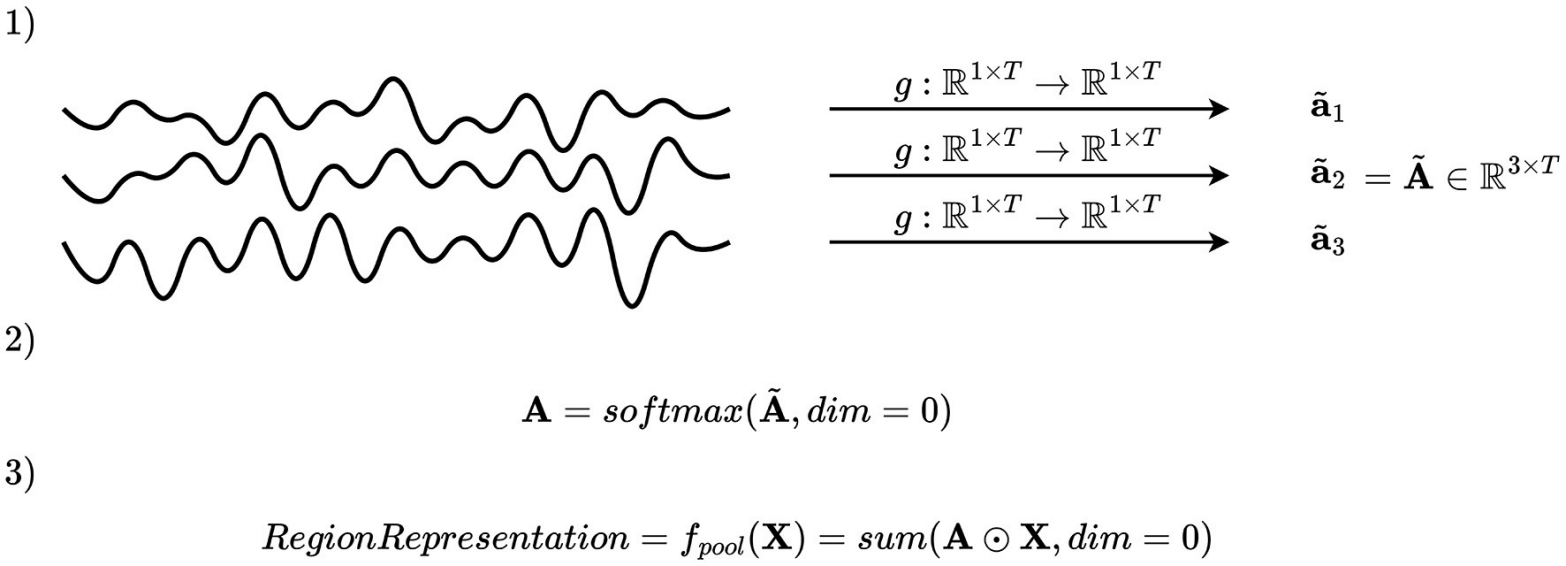
Illustration of continuous channel attention. An importance scalar is computed for every channel and time step, and the attention matrix is computed by applying softmax on a concatenation of these in the channel dimension. The attention matrix is used to compute a linear combination of the channels per time step. That is, a new linear combination is computed for each time step, allowing the pooling mechanism to shift its attention through time.

Another possible pooling mechanism is to apply continuous channel attention, which is illustrated in Figure 5. In the channel attention mechanism explained in Section 2.4.2.2, it is impossible for the model to adapt its channel attention in time. Therefore, *continuous channel attention* is implemented by defining a function *g*:ℝ^1 × *T*^ → ℝ^1 × *T*^, apply *g* to every channel, and apply softmax activation function in the channel dimension. That is, what was in Section 2.4.2.2 an attention vector of the *j*-th region in the *i*-th montage split ${\text{a}}^{\left(i,j\right)}\in \left\{\text{q}\in {\left(0,1\right)}^{\left|R_{j}^{\left(i\right)}\cap C\right|}:||\text{q}|{|}_{1}=1\right\}$ is replaced by an attention matrix ${\text{A}}^{\left(i,j\right)}\in \left\{\text{Q}\in {\left(0,1\right)}^{|R_{j}^{\left(i\right)}\cap C|\times T}:||{\text{Q}}_{:,t}|{|}_{1}=1\;\forall t\in \left\{1,2,...,T\right\}\right\}$, where all elements are positive and each column sum to 1 due to the softmax activation function. The region representation of the *j*-th region in the *i*-th montage split is followingly computed as $f_{pool}^{\left(i,j\right)}\left(\text{X},C\right)={\text{1}}^{T}\left({\text{A}}^{\left(i,j\right)}⊙{\text{X}}_{R_{j}^{\left(i\right)}\cap C}\right)$, where **1** is a vector of ones, ⊙ is the Hadamard product (element-wise multiplication), and ${\text{X}}_{R_{j}^{\left(i\right)}\cap C}\in {\mathbb{R}}^{|R_{j}^{\left(i\right)}\cap C|\times T}$ is the EEG data of the channels in $R_{j}^{\left(i\right)}\cap C$. This formulation is equivalent to applying a unique attention vector per time step.

In the experiments, an Inception network (Ismail Fawaz et al., 2020) was used as *g*. The depth of the architecture was set to two Inception modules, and the number of filters was set to two for all convolutional kernels and Inception modules. These hyperparameters were set smaller than in the original study due to high memory consumption.

##### 2.4.2.4 Region based pooling with head region

**Figure 6:**
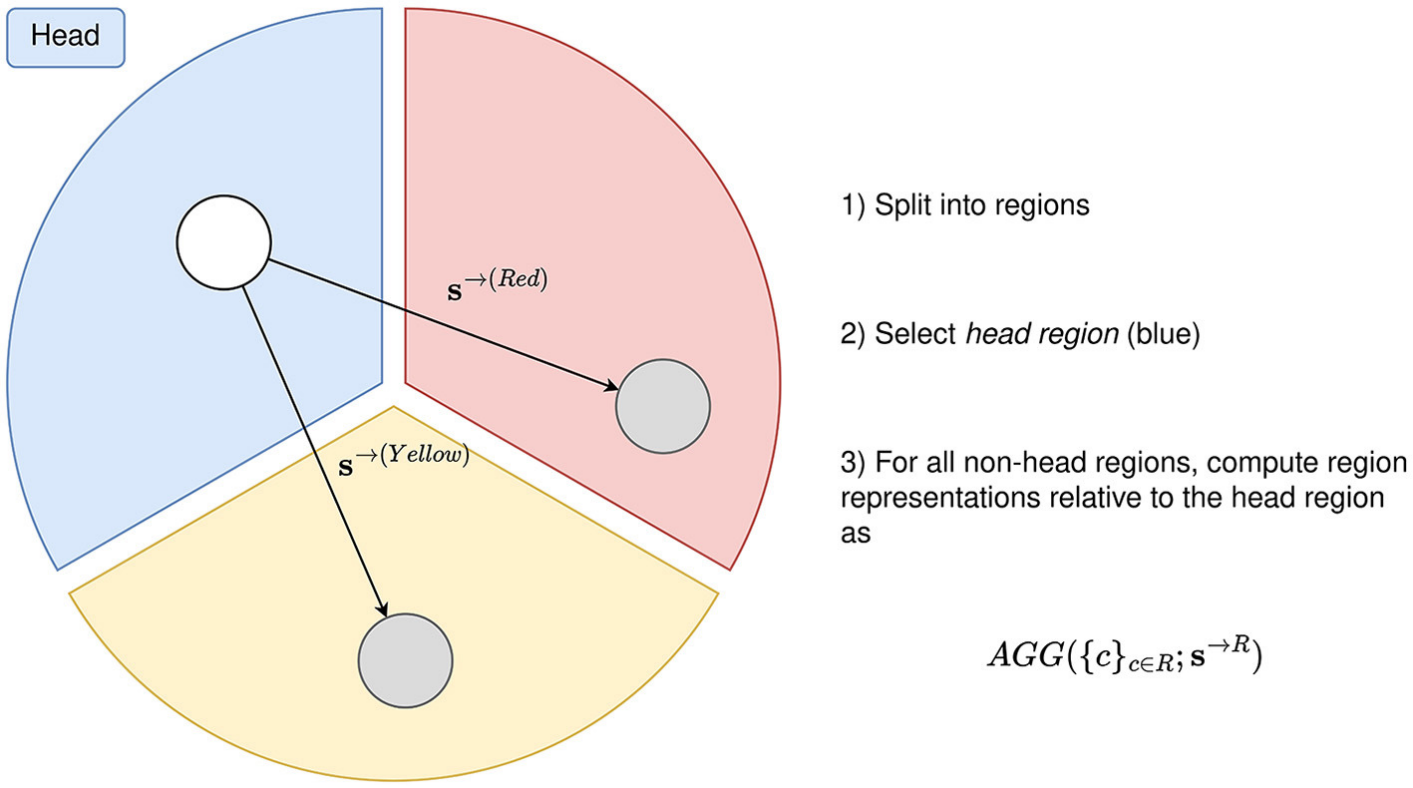
Region based pooling with a head region. The head region may influence how the channels in the non-head region should be aggregated. This is done by passing an embedding vector of the head region to the aggregation functions. By passing different embeddings to the different non-head regions, the head region is allowed to search for different features in the different spatial locations.

With the pooling mechanisms described in Sections 2.4.2.1, 2.4.2.2 and 2.4.2.3, RBP is not able to tailor the region representations based on other regions. As this may be an important property to possess, RBP can be extended to *Region Based Pooling with a Head Region*, which is illustrated in Figure 6. A *head-region* is selected, which exhibits the property of being able to influence the aggregation of channels in non-head regions.

The region representation is computed as an aggregation of the channels, given a vector embedding of the head region. For every montage split M_*i*_∈{M_1_, M_2_, ..., M_*n*_}, a head region $H^{\left(i\right)}\in M_{i}$ is selected. The region representation of all non-head regions $R_{j}^{\left(i\right)}\in M_{i}\backslash \left\{H^{\left(i\right)}\right\}$ is computed as


(1) $$\begin{array}{ll} f^{\left(i,j\right)}\left(\text{X},C\right) & =AGG^{\left(i,j\right)}\left({\text{X}}_{R_{j}^{\left(i\right)}\cap C};{\text{s}}_{i}^{\to R_{j}^{\left(i\right)}}\right)\;\forall j:R_{j}^{\left(i\right)}\in M_{i}\backslash \left\{H^{\left(i\right)}\right\}, \end{array}$$

(2) $$\begin{array}{ll} {\text{s}}_{i}^{\to R_{j}^{\left(i\right)}} & =f_{i}^{\to R_{j}^{\left(i\right)}}\left({\text{X}}_{H^{\left(i\right)}\cap C}\right), \end{array}$$

where ${\text{s}}_{i}^{\to R_{j}^{\left(i\right)}}$ is the search vector embedding of the head region *H*^(*i*)^ with relevance to region $R_{j}^{\left(i\right)}\in M_{i}\backslash \left\{H^{\left(i\right)}\right\}$, $f_{i}^{\to R_{j}^{\left(i\right)}}$ is the function mapping the channels of the head region to ${\text{s}}_{i}^{\to R_{j}^{\left(i\right)}}$, and *AGG*^(*i, j*)^ is an aggregation function. The vector embedding of the head region may thus depend on the region to compute a region representation of. The motivation of this is that the head region systematically searches for certain characteristics in the other regions, and such characteristics may depend on the given regions.

The region representation of the head region was computed as in ROCKET channel attention, introduced in Section 2.4.2.2. The search embeddings ${\text{s}}_{i}^{\to R_{j}^{\left(i\right)}}$ were computed as


(3) $$\begin{array}{ll} {\text{s}}_{i}^{\to R_{j}^{\left(i\right)}} & =\left[f_{1}^{\left(i,j\right)}\left({\text{Z}}_{H^{\left(i\right)}\cap C}\right)⊙\sigma \left(f_{2}^{\left(i,j\right)}\left({\text{Z}}_{H^{\left(i\right)}\cap C}\right)\right)\right]1, \end{array}$$

(4) $$\begin{array}{ll} f_{1}^{\left(i,j\right)}\left({\text{Z}}_{H^{\left(i\right)}\cap C}\right) & ={\text{W}}_{i,j}^{\left(1\right)}{\text{Z}}_{H^{\left(i\right)}\cap C}, \end{array}$$

(5) $$\begin{array}{ll} f_{2}^{\left(i,j\right)}\left({\text{Z}}_{H^{\left(i\right)}\cap C}\right) & ={\text{W}}_{i,j}^{\left(2\right)}{\text{Z}}_{H^{\left(i\right)}\cap C}, \end{array}$$

where σ is the softmax activation function computed in the channel dimension, ${\text{Z}}_{H^{\left(i\right)}\cap C}\in {\mathbb{R}}^{num\text{\_}kernels\cdot 2\times |H^{\left(i\right)}\cap C|}$ is a concatenation of the ROCKET features, and ${\text{W}}_{i,j}^{\left(1\right)}$ and ${\text{W}}_{i,j}^{\left(2\right)}$ are trainable weight matrices of the search embedding function of region $R_{j}^{\left(i\right)}$. The use of softmax allows the search embedding to weight the different channels in the head region differently for each ROCKET feature. The region representation of region $R_{j}^{\left(i\right)}\in M_{i}\backslash \left\{H^{\left(i\right)}\right\}$ are computed per subject as


(6) $$\begin{array}{ll} AGG^{\left(i,j\right)}\left({\text{X}}_{R_{j}^{\left(i\right)}\cap C};{\text{s}}_{i}^{\to R_{j}^{\left(i\right)}}\right) & ={\text{a}}^{\left(i,j\right)T}{\text{X}}_{R_{j}^{\left(i\right)}\cap C}, \end{array}$$

(7) $$a_{k}\;=\frac{exp\{cos(f_{1}^{\left(i,j\right)}(z_{k}),s_{i}^{\to R_{j}^{\left(i\right)}})\}}{\sum_{c\in R_{j}^{\left(i\right)}\cap C}exp\{cos(f_{1}^{\left(i,j\right)}(z_{c}),s_{i}^{\to R_{j}^{\left(i\right)}})\}},$$

with *a*_*k*_ being the elements of **a**. Note that the same embedding functions ($f_{1}^{\left(i,j\right)}$) are used on the channels of $R_{j}^{\left(i\right)}\in M_{i}\backslash \left\{H^{\left(i\right)}\right\}$ as on the channels of the head region *H*^(*i*)^. This may be beneficial, as the embeddings share the same space, and computing similarity may thus be more meaningful.

For the experiments in this study, the number of rows in the weight matrices ${\text{W}}_{i,j}^{\left(1\right)}$ and ${\text{W}}_{i,j}^{\left(2\right)}$ (and hence the dimensionality of the search vector embeddings ${\text{s}}_{i}^{\to R_{j}^{\left(i\right)}}$) were set to 64, for all *i* and *j*.

### 2.5 Experiments

All models were implemented using PyTorch (Paszke et al., 2019), version 1.10.1+cu113. The hardware used was a computer equipped with an NVIDIA GeForce RTX 3060 12GB GPU. The code is publicly available on GitHub.^2^

All models were run with learning rate set to 0.0001. The maximum number of epochs was set to 50, except for RBP with continuous channel attention, which used 20 epochs due to high time consumption. The batch size was mainly set to 16 although some models required smaller batch size due to memory constraints. The exceptions are listed in Table 1. Experiments using zero-filling and spherical spline interpolation were run with batch size set to 4, 8, 16, and 32, to ensure that potential improvements were not due to differences in batch size. Adam (Kingma and Ba, 2015) and binary crossentropy (with logits loss for improved numerical stability) were used as optimization technique and loss function, respectively.

**Table 1:** Overview of batch sizes used for grid search.

**Pooling mechanism**	**Min. number of electrodes**	**Number of montage splits**	**Batch size**
With head region	1	50	8
	2	50	8
Continuous attention	1	5	4
	2	5	4
	3	5	4
	1	10	2
	2	10	2
	3	10	2
	1	25	1
	2	25	1
	3	25	1

All RBP models not listed used a batch size of 16.

For all experiments, a 5-fold cross validation strategy was carried out. For every fold, the 4 folds not used for testing were split into training and validation 75/25. The training data was used to optimize the trainable parameters of the DL models, whereas the validation data was used to estimate what epoch to stop at. During a single fold, only the model parameters which obtained the highest area under the receiver operating characteristics curve (AUC) on the validation set (computed as the mean performance on the 32, 65, and 129-channel versions of the channel system) was used when testing on the test data fold.

To evaluate the sensitivity with respect to two new hyperparameters introduced by RBP, a grid search was made for all pooling mechanisms. The first hyperparameter was *min*_*nodes*, which is the smallest number of channels allowed in the 32-channel version of the channel system. The smaller the *min*_*nodes*, the smaller the regions are allowed to be when splitting the montage. The second hyperparameter was *num*_*montage*_*splits*, which is the number of montage splits performed. The grid search was carried out with *min*_*nodes*∈{1, 2, 3} and *num*_*montage*_*splits*∈{5, 10, 25, 50}, with the exception of RBP using continuous channel attention, which was restricted to *num*_*montage*_*splits*∈{5, 10, 25} due to memory limitations.

## 3 Results

**Figure 7:**
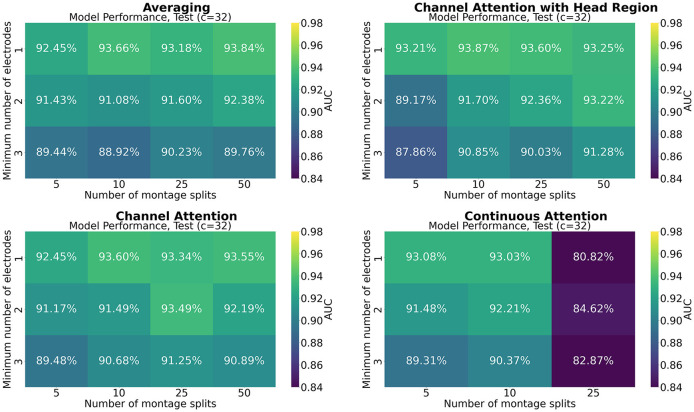
Mean performance on the channel system with 32 electrodes, as a function of number of montage splits and number of allowed electrodes in the smallest channel system.

**Figure 8:**
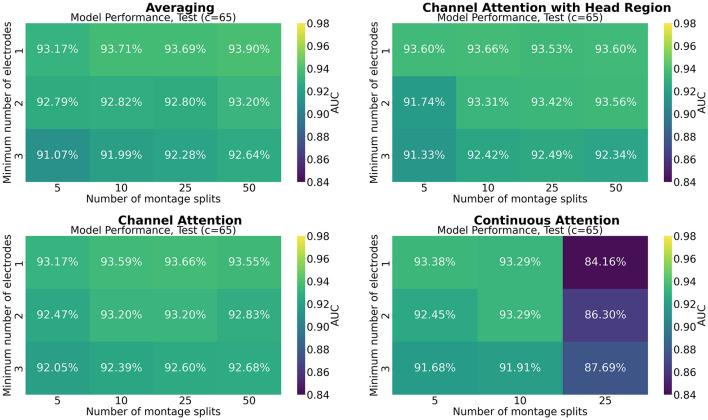
Mean performance on the channel system with 65 electrodes, as a function of number of montage splits and number of allowed electrodes in the smallest channel system.

**Figure 9:**
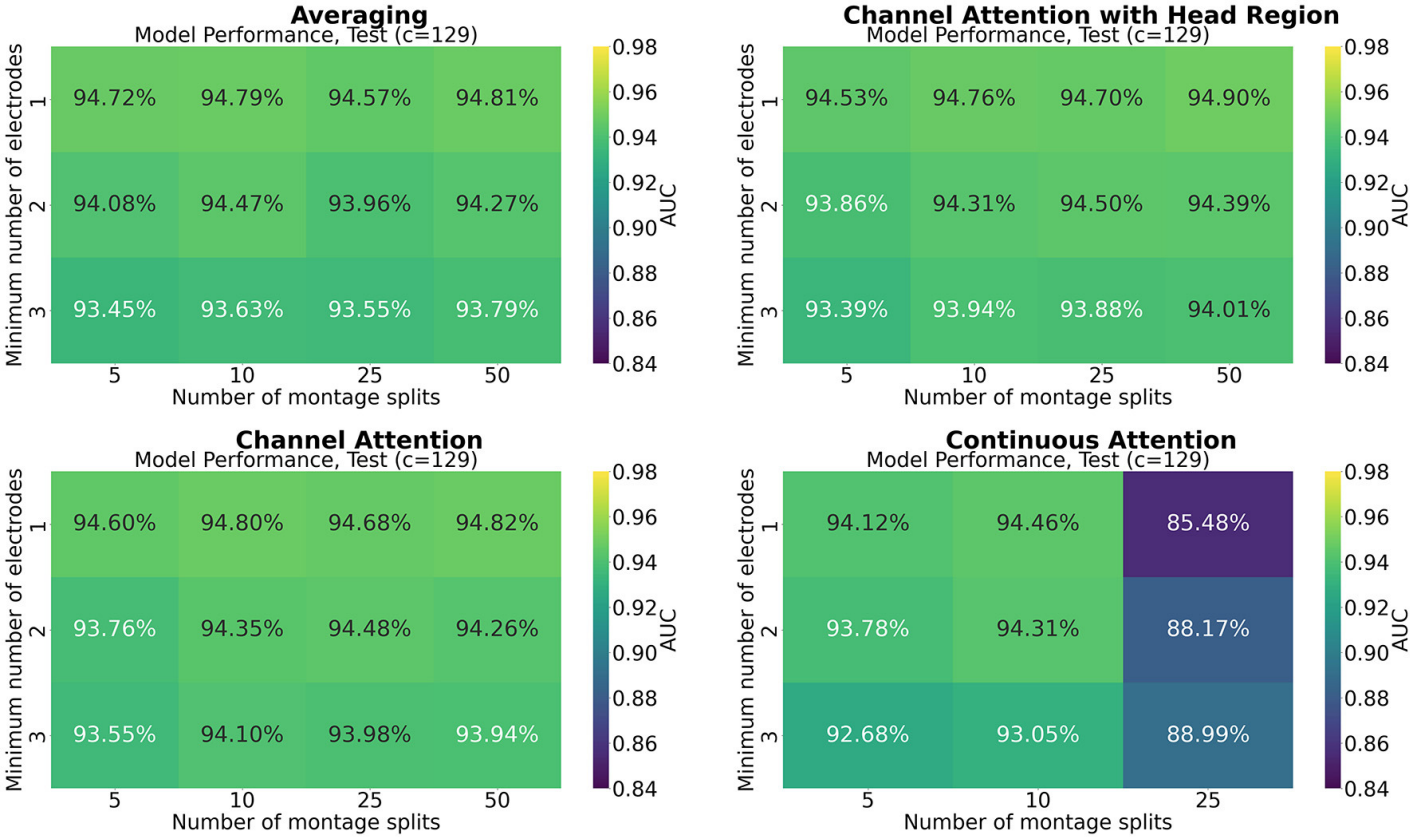
Mean performance on the channel system with 129 electrodes, as a function of number of montage splits and number of allowed electrodes in the smallest channel system.

Figures 7–9 show the results of grid search for the different pooling methods, on 32, 65, and 129 number of channels, respectively. The number in each entry represents the average performance estimate on the test sets after conducting a 5-fold cross validation. The results show that the performance is more sensitive to the selected hyperparameters for the low-resolution channel systems than the 129-channel system version. In particular, RBP seems to favor smaller regions per montage split for the downsampled channel systems.

**Figure 10:**
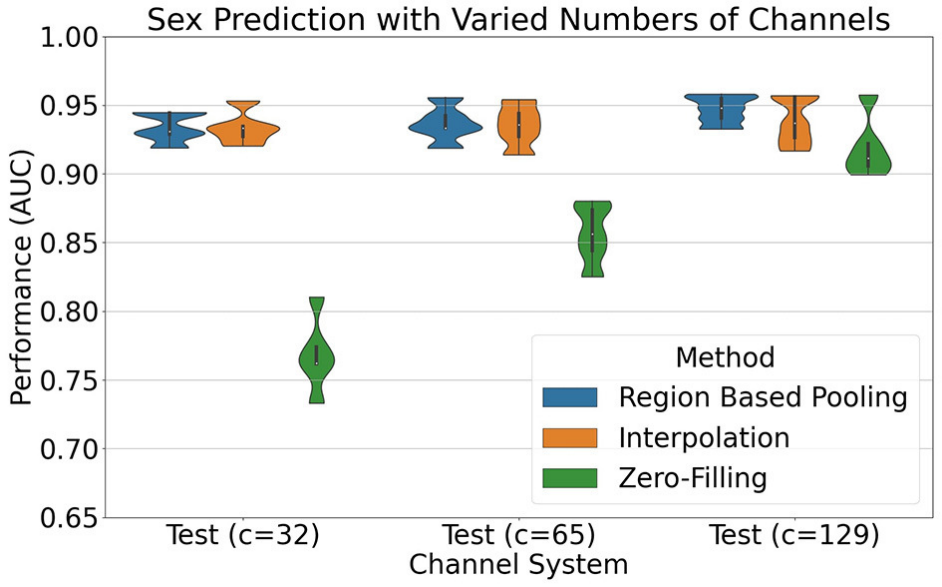
Results of sex prediction using Inception network in combination with RBP (blue), spherical spline interpolation (orange), and zero-filling (green). The splitting into 5 folds were equal for the different methods, and only the five performance estimates from the test sets are plotted. For the channel system with *c* = 129, interpolation and zero-filling are technically the same, as there are no channels to interpolate nor zero-fill. The model selection procedure, however, selected different batch sizes, and the performance differences are therefore attributed to both the model selection and differences in initialization of weights.

Figure 10 compares the performance of using RBP, spherical spline interpolation, and zero-filling. The RBP model selected used ROCKET channel attention as pooling mechanism, with number of montage splits set to 25, and *min*_*nodes* set to 1. The model selection was based on the mean validation performance on 5-fold cross validation and maximizing the mean performance on the three channel systems. The selected models using spherical spline interpolation and zero-filling used batch size set to 32 and 8, respectively, following the same model selection procedure as for RBP. For the 32-channel system version, the mean AUC values were as follows: RBP (93.34%), spherical spline interpolation (93.36%), and zero-filling (76.82%). On the 65-channel system version, the performances were RBP (93.66%), spherical spline interpolation (93.50%), and zero-filling (85.58%). Finally, the 129-channel system version produced the following results: RBP (94.68%), spherical spline interpolation (93.86%), and zero-filling (91.92%).

## 4 Discussion

### 4.1 RBP for handling a varied number of channels

RBP shows highly similar performance to spherical spline interpolation for all channel systems, as seen in Figure 10. Both RBP and spherical spline interpolation demonstrate robustness in handling a varied number of channels, as indicated by the minor performance degradation observed on the down-sampled channel systems. A potential decrease in performance when reducing the number of channels is not necessarily to be evaluated as weaknesses in these methods but may be due to a loss of information when removing channels. The objective of the methods is to handle the channel down-sampling with the smallest reduction in performance as possible although no method can restore the fully lost information. In contrast to RBP and spherical spline interpolation, zero-filling missing channels vastly reduce the performance on the lower resolution channel systems. Zero-filling is therefore not a recommended approach for handling missing channels, despite its use in, e.g., the official preprocessed version of the EEG data of the Child Mind Institute (Alexander et al., 2017).

The results from the grid searches on the different pooling mechanisms indicate that the selection of pooling mechanism was unimportant for the selected task and dataset, except for continuous channel attention for 25 number of montage splits. However, the batch size was set to 1 due to memory constraints, which is not optimal for training, and thus a strong confounder. More research is therefore needed to assess if a high number of montage splits failed in continuous channel attention due to inadequacy of the pooling mechanism or if it is solely due to the batch size. No pooling mechanism was superior to the others for all hyperparameters. A consistent trend appears to be that RBP benefits from smaller regions, as the performance on especially the channel systems with 32 and 65 channels seem to increase when the stopping criteria *min*_*nodes* decrease. This is not an unexpected finding as using smaller regions increases the spatial resolution per montage split. The current results further suggest that solely increasing the number of montage splits is insufficient when the regions are excessively large. However, as future work may include even smaller channel systems, larger regions may be beneficial from a practical point of view. Finding the optimal balance between low resolution channel systems compatibility and model performance may therefore be important for future research. However, as the model was trained only on 129 channels, the performance on the low-resolution channel systems may be increased by including them in the training data as well. For extension to the large-scale setting with multiple datasets, this is likely to be a feasible approach. Furthermore, it may be used as a data augmentation technique, in particular when the high-resolution channel system has low-resolution equivalents.

This study proposed an algorithm for splitting the EEG montage into regions although no optimization of montage splits was performed. It is likely that different EEG related problems may benefit from different montage splits. This is because the important spatial features may be task related and require higher or lower resolution of some areas. Furthermore, as only one algorithm for splitting the EEG montage into regions was tested, future work could benefit from exploring and evaluating alternative methods. Note that with the current use of regions having defined boundaries, where an electrode is either inside or not inside a region, optimizing montage splits by gradient based methods cannot work directly. This is because an infinitely small change to the boundaries of the region will either cause zero change in output or an output change of fixed size (not infinitely small, as required). The gradients would thus be either zero or infinite, making gradient based learning infeasible. Two potential solutions are further discussed in Section 4.4.2.

### 4.2 Related work

As discussed in Wei et al. (2022), limited studies has focused on generalizing DL models to handle the cross-dataset setting and a varied number of channels. A desired outcome of the BEETL competition was to develop transfer learning techniques in the cross-dataset setting (Wei et al., 2022). However, the top three entries selected simple methods to handle a varied number of channels and the difference in channel locations; channel removal, dataset removal, or both. Furthermore, to handle a varied number of channels in the pre-training and downstream training, Kostas et al. (2021) mapped all datasets to 19 channels, and in that process, sacrificed a considerable part of the data for several of the datasets used for downstream training. However, research from clinical neurology suggests that certain characteristics require high-density EEG with an increased number of channels (Kuhnke et al., 2018; Hatlestad-Hall et al., 2023). The feasibility of downsampling the spatial resolution may therefore be limited to only a subset of EEG-related tasks.

Li and Metsis (2022) developed SPP-EEGNET, an architecture designed for inter-dataset transfer learning, and is compatible with a varied number of channels. However, SPP-EEGNET pools the feature maps by spatial pyramid pooling (SPP) (He et al., 2014) after convolutions have been applied channel-wise. Cross-channel patterns can therefore not be extracted by the convolutional module of SPP-EEGNET as the receptive field of the feature maps are bounded to their respective single channel. Such cross-channel patterns may only be extracted by the fully connected module, after applying the SPP layer. As the success of signal processing is mostly attributed to the convolutional module, this approach may be sub-optimal. Furthermore, many existing DL architectures for EEG data apply 1D convolutions across channels, hindering its application to many of the currently existing architectures. This contrasts with RBP, which is compatible with any DL model for multivariate time series classification/regression.^3^ This is beneficial, as the current high-performing models from literature may apply RBP with ease (simply use RBP as the initial layer), meaning the accumulated research and development on DL architectures over time is respected. Furthermore, it offers a simple solution for working on the cross-dataset and cross-channel system setting in the future. Note also that although this study represented the EEG data as time series, using other representations such as power spectral density or operating on wavelet transformed images are popular choices of input to DL models. RBP is indeed compatible with such representations although the pooling mechanisms must be tailored to fit the input domain. Finally, the pooling in RBP is performed based on the spatial positions of the electrodes, whereas SPP-EEGNET does not precisely specify how the feature maps of the different channels were merged. If the pooling is made only by the data matrix **X** [as if it was an image, following the original SPP-net (He et al., 2014)], then inconsistency in which channels end up in which spatial region will occur.

### 4.3 Limitations of the study

A limitation of this study is its reliance on a single dataset and classification problem, which may restrict the generalizability of the findings. In particular, the size of the dataset was larger than what is commonly available for EEG datasets with more clinically relevant labels. When the total number of region representations exceeds the number of channels in a given channel system, RBP effectively expands the dimensionality of the data. This is especially the case when the regions are small, and the number of montage splits are many. For smaller datasets in particular, this may lead to an increased risk of overfitting. The generalizability of the results to smaller datasets, and in particular, the effect of the hyperparameters *min*_*nodes* and *num*_*montage*_*splits* is therefore poorly investigated. While testing the methods on sex classification allowed for a large dataset with low chance of false labeling, its clinical utility is low. Thus, classification/regression problems with higher clinical relevance should be considered in the future. Furthermore, only a single model (Inception network) was used in combination with the three different methods for handling a varied number of electrodes. Although Inception network is an effective DL model for multivariate time series analysis, generalization to other models was not assessed. This is needed due to the high number of DL models used for EEG analysis. Finally, hardware limitations constrained the training of all RBP models using the same batch size, potentially reducing the performance of the models with smaller batch size. By testing with more models, datasets, and classification/regression problems, the relevance of the methods will thus be better addressed. In particular, to fully explore the potential and relevance of the investigated methods, experiments including datasets with even smaller numbers of EEG channels, such as 19 or 25, are required.

### 4.4 Future work

#### 4.4.1 Pooling mechanisms and hyperparameters

The use of features as computed in ROCKET, and a single linear layer to compute the importance score of a channel, provides a light-weight method for computing channel attention. It was selected based on its light-weightedness as the sole purpose of the pooling mechanism is to compute coefficients of a linear combination. Furthermore, the extracted ROCKET features could be pre-computed prior to training, making it a pragmatic choice for run-time efficiency. Using more powerful DL models was hypothesized to be unnecessary and overpowered for such a task although in the absence of proper experimental results in this regard, final conclusions cannot be drawn. Using pooling mechanisms which selects not only the channels of interest but also the frequency bands of interest is a possible future direction.

All pooling mechanisms used in the experiments were compatible with a single channel per region. This is the case, e.g., for computing channel attention using ROCKET features, as the function *g* for computing the importance score of a channel only uses the features of that very channel. Future work may attempt to define pooling mechanisms which require more than one channel per region. This may be accomplished by e.g. extending the input domain and output range of *g* to $g:{\mathbb{R}}^{p_{in}\times T}\to {\mathbb{R}}^{p_{out}}$, where *p*_*in*_ is the lower bound of accepted number of channels in a region, and *p*_*out*_ is the number of output features per application of *g*. However, as this may either require larger regions (which by the current results does not appear to be favorable) or lead to incompatibility with the low-resolution channel systems, it is important to determine if the potential benefits outweigh the drawbacks in future research.

All experiments in this study merged the different montage splits by concatenation directly after the pooling was made. Another approach could be to apply convolutional modules separately on the montage splits, prior to merging them. Furthermore, other approaches such as summation, averaging, or alternating between applying convolution and adding a montage split such as skip connections, are examples of other possible pooling strategies. In particular, merging montage splits by skip connections and using dynamic neural networks (Han et al., 2022) to e.g. perform a sample or channel system conditioned number of montage splits by early exiting or layer skipping is a possible future direction. By using dynamic architectures, more montage splits could be used on the high-resolution channel systems, and fewer montage splits could be used on the low-resolution channel systems. Furthermore, montage splits with small regions could be used on high-resolution channel systems only, possibly alleviating the here observed trade-off between performance and low-resolution compatibility.

#### 4.4.2 Splitting into regions

While the current study did not perform any optimization of the splitting of the EEG montage into regions, two possible solutions which may be explored in the future are (1) use other techniques for optimizing. One approach could be to generate many splits and apply sparsity. (2) Introduce soft regions, where electrodes are assigned a non-binary weight to its presence in the region. A region could e.g. be represented as a Gaussian, where the mean and standard deviation are treated as trainable parameters. The influence of a specific channel on a region representation would be determined by both an importance score calculated from a function *g* operating on the time series, and its spatial importance given the properties of the region (e.g., mean and standard deviation).

#### 4.4.3 Training strategies with large amounts of data

A major motivation behind RBP is to enable the use of multiple and heterogeneous datasets with a varied number of channels for different training strategies. Large-scaled use of multiple datasets should be tested for methods such as pre-training (e.g., transfer learning or self-supervised learning), representation learning (e.g., self-supervised or unsupervised learning), and simply using more datasets if the same targets are available. Fixing different electrode arrays and using spherical spline interpolation in the case of varied channel systems across the datasets, should be used as baselines.

For the AI-Mind project, this may be of high relevance for both improving the DL model performance and generalization. While the project aims at collecting a dataset comprised of 1,000 participants and possibly expanding this with synthetic data, this is not guaranteed to be sufficient for DL models. Improving data efficiency and model performance by the abovementioned training strategies may be enhanced by enabling them in the cross-channel system setting. Furthermore, data collection from four different countries and five different clinical sites is likely to mitigate bias to some extent. However, its sufficiency is difficult to address a priori. Two arguments against, are that (1) all clinical sites are situated in European countries, and (2) the hardware for EEG recordings are the same. Thus, by applying the abovementioned training strategies to heterogeneous datasets, the ability of the DL models to generalize across populations and hardware may be improved.

## 5 Conclusion

Region based pooling was introduced for deep learning models to handle a varied number of EEG channels. Furthermore, its adequacy in maintaining performance when downsampling the channel system was experimentally demonstrated. Grid search was used to assess the effect of two new hyperparameters, which relates to the size of the regions and the number of montage splits. Several pooling mechanisms were introduced and tested, yielding highly similar results. Region based pooling obtained similar results to spherical spline interpolation, and superior results to zero-filling missing channels when downsampling the channel system to 65 and 32 channels. Zero-filling missing channels is therefore not a recommended method for handling a varied number of channels. Future work includes applying region based pooling on multiple and heterogeneous datasets with different EEG channel systems. In particular, large-scale pre-training and representation learning in combination with region based pooling will be investigated.

## Data availability statement

The original contributions presented in the study are included in the article/supplementary material, further inquiries can be directed to the corresponding author.

## Ethics statement

Ethical approval was not required for the study involving humans in accordance with the local legislation and institutional requirements. Written informed consent to participate in this study was not required from the participants or the participants' legal guardians/next of kin in accordance with the national legislation and the institutional requirements.

## Author contributions

TT: Conceptualization, Data curation, Formal analysis, Methodology, Visualization, Writing—original draft, Writing—review & editing. MT: Data curation, Validation, Writing—review & editing. AP: Writing—review & editing. CH-H: Data curation, Supervision, Writing—review & editing, Project administration. AY: Supervision, Writing—review & editing. HH: Methodology, Supervision, Validation, Writing—review & editing. IH: Funding acquisition, Project administration, Resources, Supervision, Writing—review & editing.

## Footnotes

^1^ https://github.com/hatlestad-hall/prep-childmind-eeg

^2^ https://github.com/thomastveitstol/RegionBasedPoolingEEG

^3^Although one requirement is differentiability, if the pooling mechanism has parameters to be optimized as part of the gradient based learning.

## References

[B1] Abo Alzahab N. Apollonio L. Di Iorio A. Alshalak M. Iarlori S. Ferracuti F.. (2021). Hybrid deep learning (hdl)-based brain-computer interface (bci) systems: a systematic review. Brain Sci. 11, 75. 10.3390/brainsci11010075 33429938 PMC7827826

[B2] Ahmad I. Wang X. Zhu M. Wang C. Pi Y. Khan J. A.. (2022). Eeg-based epileptic seizure detection via machine/deep learning approaches: a systematic review. Intell. Neurosci. 2022, 6486570. 10.1155/2022/6486570 PMC9232335 35755757

[B3] Alexander L. M. Escalera J. Ai L. Andreotti C. Febre K. Mangone A.. (2017). An open resource for transdiagnostic research in pediatric mental health and learning disorders. Sci. Data 4, 170181. 10.1101/149369 29257126 PMC5735921

[B4] Banville H. Chehab O. Hyvarinen A. Engemann D.-A. Gramfort A. (2021). Uncovering the structure of clinical EEG signals with self-supervised learning. J. Neural Eng. 18, 46020. 10.1088/1741-2552/abca18 33181507

[B5] Chen R. J. Wang J. J. Williamson D. F. K. Chen T. Y. Lipkova J. Lu M. Y.. (2023). Algorithmic fairness in artificial intelligence for medicine and healthcare. Nat. Biomed. Eng. 7, 719–742. 10.1038/s41551-023-01056-8 37380750 PMC10632090

[B6] de Bardeci M. Ip C. T. Olbrich S. (2021). Deep learning applied to electroencephalogram data in mental disorders: a systematic review. Biol. Psychol. 162, 108117. 10.1016/j.biopsycho.2021.108117 33991592

[B7] de Cheveigné A. (2020). Zapline: a simple and effective method to remove power line artifacts. Neuroimage 207, 116356. 10.1016/j.neuroimage.2019.116356 31786167

[B8] Delorme A. Makeig S. (2004). Eeglab: an open source toolbox for analysis of single-trial eeg dynamics including independent component analysis. J. Neurosci. Methods 134, 9–21. 10.1016/j.jneumeth.2003.10.009 15102499

[B9] Dempster A. Petitjean F. Webb G. I. (2020). ROCKET: exceptionally fast and accurate time series classification using random convolutional kernels. Data Min. Knowl. Discov. 34, 1454–1495. 10.1007/s10618-020-00701-z

[B10] Farsi L. Siuly S. Kabir E. Wang H. (2021). Classification of alcoholic eeg signals using a deep learning method. IEEE Sens. J. 21, 3552–3560. 10.1109/JSEN.2020.3026830

[B11] Goodfellow I. Pouget-Abadie J. Mirza M. Xu B. Warde-Farley D. Ozair S.. (2014). “Generative adversarianets,” in Advances in Neural Information Processing Systems, Vol. 27, eds Z. Ghahramani, M. Welling, C. Cortes, N. Lawrence, and K. Weinberger (Curran Associates, Inc.).

[B12] Grieves M. Vickers J. (2017). Digital Twin: Mitigating Unpredictable, Undesirable Emergent Behavior in Complex Systems (Cham: Springer International Publishing), 85–113.

[B13] Han Y. Huang G. Song S. Yang L. Wang H. Wang Y. (2022). Dynamic neural networks: a survey. IEEE Trans. Pattern Anal. Mach. Intell. 44, 7436–7456. 10.1109/TPAMI.2021.3117837 34613907

[B14] Hatlestad-Hall C. Bruña R. Liljeström M. Renvall H. Heuser K. Taubøll E.. (2023). Reliable evaluation of functional connectivity and graph theory measures in source-level eeg: how many electrodes are enough? Clin. Neurophysiol. 150, 1–16. 10.1016/j.clinph.2023.03.002 36972647

[B15] He K. Zhang X. Ren S. Sun J. (2014). “Spatial pyramid pooling in deep convolutional networks for visual recognition,” in Computer Vision-ECCV 2014, eds D. Fleet, T. Pajdla, B. Schiele, and T. Tuytelaars (Cham: Springer International Publishing), 346–361.26353135

[B16] Hendrycks D. Lee K. Mazeika M. (2019). “Using pre-training can improve model robustness and uncertainty,” in Proceedings of the 36th International Conference on Machine Learning, volume 97 of Proceedings of Machine Learning Research, eds K. Chaudhuri, and R. Salakhutdinov (PMLR), 2712–2721.

[B17] Hinton G. (2018). Deep learning—a technology with the potential to transform health care. JAMA 320, 1101–1102. 10.1001/jama.2018.11100 30178065

[B18] Houssein E. Hamad A. Ali A. (2022). Human emotion recognition from eeg-based brain–computer interface using machine learning: a comprehensive review. Neural Comp. Appl. 34, 12527–12557. 10.1007/s00521-022-07292-4

[B19] Ismail Fawaz H. Lucas B. Forestier G. Pelletier C. Schmidt D. F. Weber J.. (2020). Inceptiontime: Finding alexnet for time series classification. Data Min. Knowl. Discov. 34, 1936–1962. 10.1007/s10618-020-00710-y

[B20] Kelly C. J. Karthikesalingam A. Suleyman M. Corrado G. King D. (2019). Key challenges for delivering clinical impact with artificial intelligence. BMC Med. 17, 195. 10.1186/s12916-019-1426-2 31665002 PMC6821018

[B21] Kingma D. P. Ba J. (2015). “Adam: a method for stochastic optimization,” in 3rd International Conference on Learning Representations, ICLR 2015, Conference Track Proceedings, eds Y. Bengio, and Y. LeCun (San Diego, CA), 7–9.

[B22] Kostas D. Aroca-Ouellette S. Rudzicz F. (2021). Bendr: using transformers and a contrastive self-supervised learning task to learn from massive amounts of EEG data. Front. Hum. Neurosci. 15, 653659. 10.3389/fnhum.2021.653659 34248521 PMC8261053

[B23] Kuhnke N. Schwind J. Dümpelmann M. Mader M. Schulze-Bonhage A. Jacobs J. (2018). High frequency oscillations in the ripple band (80-250 hz) in scalp EEG: Higher density of electrodes allows for better localization of the seizure onset zone. Brain Topogr. 31, 1059–1072. 10.1007/s10548-018-0658-3 29980967

[B24] LeCun Y. Bengio Y. Hinton G. (2015). Deep learning. Nature 521, 436-444. 10.1038/nature14539 26017442

[B25] Li X. Metsis V. (2022). “SPP-EEGNET: an input-agnostic self-supervised EEG representation model for inter-dataset transfer learning,” in Proceedings of the 18th International Conference on Computing and Information Technology (IC2IT 2022) (Cham: Springer International Publishing), 173–182. 10.1007/978-3-030-99948-3_17

[B26] Lotte F. Bougrain L. Cichocki A. Clerc M. Congedo M. Rakotomamonjy A.. (2018). A review of classification algorithms for EEG-based brain–computer interfaces: a 10 year update. J. Neural Eng. 15, 031005. 10.1088/1741-2552/aab2f2 29488902

[B27] Mohammed R. Miften F. George L. (2022). Driver drowsiness detection methods using eeg signals: a systematic review. Comp. Methods Biomech. Biomed. Eng. 26, 1–13. 10.1080/10255842.2022.2112574 35983784

[B28] Oh S. L. Vicnesh J. Ciaccio E. J. Yuvaraj R. Acharya U. R. (2019). Deep convolutional neural network model for automated diagnosis of schizophrenia using eeg signals. Appl. Sci. 9, 2870. 10.3390/app9142870

[B29] Paszke A. Gross S. Massa F. Lerer A. Bradbury J. Chanan G.. (2019). PyTorch: An Imperative Style, High-Performance Deep Learning Library. Red Hook, NY: Curran Associates Inc.

[B30] Perrin F. Pernier J. Bertrand O. Echallier J. (1989). Spherical splines for scalp potential and current density mapping. Electroencephalogr. Clin. Neurophysiol. 72, 184–187. 10.1016/0013-4694(89)90180-6 2464490

[B31] Rajpurkar P. Chen E. Banerjee O. Topol E. J. (2022). Ai in health and medicine. Nat. Med. 28, 31-38. 10.1038/s41591-021-01614-0 35058619

[B32] Roy Y. Banville H. Albuquerque I. Gramfort A. Falk T. H. Faubert J. (2019). Deep learning-based electroencephalography analysis: a systematic review. J. Neural Eng. 16, 051001. 10.1088/1741-2552/ab260c 31151119

[B33] Ruiz A. Flynn M. Large J. Middlehurst M. Bagnall A. (2021). The great multivariate time series classification bake off: a review and experimental evaluation of recent algorithmic advances. Data Min. Knowl. Discov. 35, 1–49. 10.1007/s10618-020-00727-3 33679210 PMC7897627

[B34] Stam C. Jones B. Nolte G. Breakspear M. Scheltens P. (2006). Small-world networks and functional connectivity in Alzheimer's disease. Cereb. Cortex 17, 92–99. 10.1093/cercor/bhj127 16452642

[B35] Stancin I. Cifrek M. Jovic A. (2021). A review of eeg signal features and their application in driver drowsiness detection systems. Sensors 21, 3786. 10.3390/s21113786 34070732 PMC8198610

[B36] Stephan B. C. M. Pakpahan E. Siervo M. Licher S. Muniz-Terrera G. Mohan D.. (2020). Prediction of dementia risk in low-income and middle-income countries (the 10/66 study): an independent external validation of existing models. Lancet Global Health 8, e524–e535. 10.1016/S2214-109X(20)30062-0 32199121 PMC7090906

[B37] Wei X. Faisal A. A. Grosse-Wentrup M. Gramfort A. Chevallier S. Jayaram V.. (2022). “2021 beetl competition: advancing transfer learning for subject independence & heterogenous eeg data sets,” in Proceedings of the NeurIPS 2021 Competitions and Demonstrations Track, volume 176 of Proceedings of Machine Learning Research, eds D. Kiela, M. Ciccone, and B. Caputo (PMLR), 205–219.

[B38] Yasin S. Hussain S. A. Aslan S. Raza I. Muzammel M. Othmani A. (2021). Eeg based major depressive disorder and bipolar disorder detection using neural networks:a review. Comput. Methods Programs Biomed. 202, 106007. 10.1016/j.cmpb.2021.106007 33657466

